# Endocrine manifestations related to inherited metabolic diseases in adults

**DOI:** 10.1186/1750-1172-7-11

**Published:** 2012-01-28

**Authors:** Marie-Christine Vantyghem, Dries Dobbelaere, Karine Mention, Jean-Louis Wemeau, Jean-Marie Saudubray, Claire Douillard

**Affiliations:** 1 Service d'Endocrinologie et Maladies Métaboliques, 1, Rue Polonovski, Hôpital C Huriez., Centre Hospitalier Régional et Universitaire de Lille, 59037 Lille cedex, France; 2 Centre de Référence des Erreurs Innées du Métabolisme -Hôpital Jeanne de Flandres, Centre Hospitalier Régional et Universitaire de Lille, 59037 Lille cedex, France; 3 Département des Maladies Métaboliques, Fédération des Maladies du Système Nerveux, Hôpital Pitié-Salpêtrière, Assistance Publique Hôpitaux de Paris et Université Pierre et Marie Curie (Paris VI), France

**Keywords:** Inborn errors of metabolism, endocrine dysfunction, hypogonadism, diabetes mellitus, thyroid dysfunction, hypopituitarism, adrenal failure, hypoparathyroidism

## Abstract

Most inborn errors of metabolism (IEM) are recessive, genetically transmitted diseases and are classified into 3 main groups according to their mechanisms: cellular intoxication, energy deficiency, and defects of complex molecules. They can be associated with endocrine manifestations, which may be complications from a previously diagnosed IEM of childhood onset. More rarely, endocrinopathies can signal an IEM in adulthood, which should be suspected when an endocrine disorder is associated with multisystemic involvement (neurological, muscular, hepatic features, etc.). IEM can affect all glands, but diabetes mellitus, thyroid dysfunction and hypogonadism are the most frequent disorders. A single IEM can present with multiple endocrine dysfunctions, especially those involving energy deficiency (respiratory chain defects), and metal (hemochromatosis) and storage disorders (cystinosis). Non-autoimmune diabetes mellitus, thyroid dysfunction and/or goiter and sometimes hypoparathyroidism should steer the diagnosis towards a respiratory chain defect. Hypogonadotropic hypogonadism is frequent in haemochromatosis (often associated with diabetes), whereas primary hypogonadism is reported in Alström disease and cystinosis (both associated with diabetes, the latter also with thyroid dysfunction) and galactosemia. Hypogonadism is also frequent in X-linked adrenoleukodystrophy (with adrenal failure), congenital disorders of glycosylation, and Fabry and glycogen storage diseases (along with thyroid dysfunction in the first 3 and diabetes in the last). This is a new and growing field and is not yet very well recognized in adulthood despite its consequences on growth, bone metabolism and fertility. For this reason, physicians managing adult patients should be aware of these diagnoses.

## Introduction

Inborn errors of metabolism (IEM) are rare genetic diseases, which usually have a recessive mode of inheritance. They are classified into 3 main groups according to their mechanisms: cellular intoxication, energy deficiency, and degradation or synthesis defects of complex molecules [1]. This is a new and growing field in adulthood and is not yet very well known.

Endocrine manifestations can be a complication of a previously diagnosed IEM. More rarely they may signal the presence of a new IEM, mostly those involving glucose metabolism and presenting with hypoglycaemia (see the related article for review). Nevertheless all types of endocrine disorders have been described in IEM (Table 1), and the endocrinologist should be aware of them when an endocrine disorder is associated with multisystem involvement, for example with neurological, muscular and/or hepatic features.

**Table 1:** Endocrine manifestations of inborn errors of metabolism in adults

IEM General classification	IEM associated with endocrine manifestations and *most important mechanism*	ENDOCRINE MANIFESTATIONS					
		
		Diabetes	Dysthyroidism	Hypopara- thyroidism	Adrenal failure	Hypogonadism	Hypopituitarism
**INTOXICATION** **Metal intoxication** Haemochromatosis Aceruleoplasminemia Wilson's **Sugar intolerances** Galactosaemia **Most organic acidurias**	**Haemochromatosis** *Iron storage*	**Diabetes** **10%**	< 1%	< 1%	< 1%	**Hypogonadotropic** **5-10%**	< 1% except for acquired iron overload and hypogonadism
	
	**Aceruloplasminemia** *Iron storage*	**Diabetes**					rare
	
	**Wilson's disease** *Copper storage*			Hypoparathyroidism rare			
	
	**Galactosaemia** *Galactose metabolites*					Hypergonadotropic in female	**Short stature**
	
	**Organic aciduria** *Organic acids*	Possible ketoacidosis pancreatitis					

**ENERGY DEFECT** **Mitochondrial disorders**: Respiratory chain disorders Fatty acid oxidation defects	Respiratory chain defect Deficient energy production	**Diabetes**	**All types of thyroid dysfunction**	Hypoparathyroidism rare	Subclinical adrenal failure	**Hypogonadotropic**	**Hypopituitarism** **Short stature** **30 to 50%**
	
	LCHAD Deficient energy production			**Hypo-** **parathyroidism**			

**Cytoplasmic energy defects** Disorders of glycogen metabolism	**Glycogenosis** Glycogen storage (type I, III)	Diabetes	**Thyroid dysfunction** (type Ib)			**Mixed or undetemined** PCOS (type I)	

***COMPLEX MOLECULES*** ***Peroxisomal disorders***	**X-linked adreno-leukodystrophy** **Perrault syndrome** VLCFA accumulation				**Adrenal failure**	**Mixed or undetemined** **(mainly hypergonadotropic)**	

**Lysosomal disorders**	**Fabry disease** Globoside storage in lysosomes		**Subclinical** **hypothyroidism**		Subclinical adrenal failure	Infertility	
	
	**Cystinosis** Cystine in lysosomes	**Diabetes**	**Hypothyroidism** **75%**			Hypergonadotropic	**Short stature** **30 to 50%**

**Disorders of intracellular **trafficking and processing such as Congenital disorders of glycosylation Inborn errors of cholesterol synthesis.	**CDG I** Abnormal glycosylated proteins		Congenital hypothyroidism			**Mixed or undetermined** **mainly hypergonadotropic**	
	
	**Multisystemic triglyceride storage disease** Triglyceride storage in endoplasmic reticulum		Thyroid dystrophy				
	
	**Type 1 hyperoxaluria** Oxalate		**Hypothyroidism**				Advanced bone age
	
	**Type B Niemann-Pick disease** Sphingolipid storage in lysosomes				Partial rare		Short stature

***TRANSPORTER*** ***DEFECT***	**Rogers syndrome:** **Thiamine-sensitive megaloblastic anaemia** Defective ATP production in ß cells	**Diabetes**					
	
	**MCT8 deficiency** Defective T3 transport in neurons		**High blood T3**				
	
	**Alström syndrome**	**Diabetes**	Hypothyroidism			Hyper- and hypogonadotropic (men) PCOS in women	Rare hypopituitarism Initial advanced bone age
	
	Selenoprotein deficiency disorder		Low T3-High T4			Oligospermia	Short stature

CDG: congenital disorders of glycosylation; IEM: inborn errors of metabolism; LCHAD: long-chain 3-hydroxyacyl-CoA dehydrogenase deficiency; MCT8: Monocarboxylate transporter; PCOS: polycystic ovary syndrome; T3: triiodothyronine; T4: thyroxine; VLCFA: Very long chain fatty acids.

Many endocrine manifestations in IEM might go unrecognized, especially with regard to subclinical dysfunctions. Otherwise, the long-term consequences of IEM on growth, bone metabolism and fertility have not been prospectively investigated.

When fertility is maintained, all pregnancies in patients with a previously diagnosed IEM should be carefully planned and monitored, especially those with dietary management, such as with phenylketonuria. Preconception screening for hormone deficiency, especially hypothyroidism, is recommended in most IEM, as is investigation for gestational diabetes. Genetic counseling should be provided, even though most IEMs, except for mitochondrial diseases, are inherited in an autosomal recessive manner. Prenatal testing for high-risk pregnancies may be discussed if the disease-causing mutations in the family are known.

The aim of this review is to identify the main IEMs in adults that can be revealed or complicated by an endocrine disorder other than hypoglycaemia.

## Why endocrine disorders in IEM?

Hormones play an essential role in the coordination of complex functions such as growth, reproduction, metabolism and energy homeostasis. Hormonal disturbance could contribute to IEM consequences in adulthood. The endocrine system also has paracrine and autocrine functions and is intimately connected with nervous and immune networks by neuromediators and cytokines.

Hormones are classified according to three types: 1) peptide hormones (insulin, parathyroid hormone, pituitary hormones), 2) hormones synthesized from a single amino-acid (catecholamines, thyroid hormones), all derived from tyrosine; and 3) steroid hormones (adrenal and gonadal hormones), all synthesized from cholesterol. Peptide hormones and catecholamines are hydrosoluble and act through different kinds of membrane receptors, the activation of which requires energy. In contrast, steroid and thyroid hormones are liposoluble, circulate bound to specific transporter proteins and are able to cross the cell membrane before acting through cytoplasmic or nuclear receptors. On the whole, the pathophysiology of endocrine dysfunctions in IEM remains poorly understood. IEM can affect all glands, both in their structure and their secretion, but diabetes mellitus, thyroid dysfunction and hypogonadism are the most commonly associated disorders.

The three mechanisms known to interfere with hormone metabolism may act by 1) ruining the gland structure through progressive accumulation of toxic substances like metal [iron in haemochromatosis (> 1/1000, ORPHA139498)], cystine [cystinosis (< 1-9/1000000, ORPHA213)] or complex molecules [sphingolipids, glycogen, galactose, abnormal glycosylated compounds, or very long chain fatty acids (VLCFA)]; 2) directly or indirectly disturbing the energy availability required for hormone synthesis (as in respiratory chain disorders) or hormone release (as in underproduction of insulin-causing diabetes in Rogers syndrome (< 1-9/1000000, ORPHA49827); or 3) preventing correct hormone biosynthesis in organelles or hormone transport into the target organ, as in cerebral monocarboxylate 8 (MCT8) defect, in which the thyroid hormone cannot cross the blood brain barrier.

### Intoxication diseases: Endocrine consequences mainly in haemochromatosis and galactosemia (< 1-9/100000, ORPHA352) (Table 1)

No obvious endocrine consequences have been described so far in aminoacidopathies, urea cycle disorders or organic acidurias.

In haemochromatosis the pancreas and gonads are the most frequently affected glands with an accumulation of iron. Clinical manifestations can be prevented or improved through treatment. In contrast, hypogonadism in female patients with galactosemia, the mechanism of which is not known, is a unique endocrine complication. It is nearly constant in most cases despite early prospective treatment. Diagnosis is easy and relies on blood and urine analyses.

### Disorders of energy metabolism: Frequent multi-endocrine involvement (Table 1)

Respiratory chain defects and glycogen storage diseases (GSD) are associated with the highest number of endocrine anomalies. Energy deficient production in the former (in which all glands can be involved) and glycogen deposits and secondary glycosylation disturbances in the latter (in which only the pancreas and ovaries are affected) are the most probable (but not demonstrated) mechanisms involved in these disorders.

Diagnosis is difficult and relies on function tests; enzymatic analyses requiring biopsies or cell culture; and molecular analyses.

### Organelle disorders involving the synthesis or degradation of complex molecules: Main endocrine consequences on liposoluble hormones (Table 1)

Fabry disease (< 1-9/100000, ORPHA324) and cystinosis (< 1-9/1000000, ORPHA213), both lysosomal, X-linked adrenoleukodystrophy (X-ALD) (1-9/100000, ORPHA43), peroxisomal, and congenital disorders of glycosylation (CDG) (< 1/1000000, ORPHA137), both related to endoplasmic reticulum and Golgi dysfunction, are the most frequent IEM responsible for endocrine manifestations in this group (Table 1). In cystinosis, lysosomal cystine accumulation may cause diabetes, hypogonadism, hypothyroidism and short stature without a clear mechanism. These complications are only partially prevented or improved with cysteamine treatment. In Fabry disease, subclinical dysthyroidism is frequent and seems to respond to enzyme therapy. Adrenal failure is an almost constant finding in X-ALD and is caused by defective catabolism resulting in VLCFA accumulation, which interferes with steroid hormone synthesis. Hypogonadism may also be observed in X-ALD and Perrault syndrome (< 1/1000000, ORPHA2855), in which a defect in peroxisomal VLCFA oxidation has been recently described. Abnormal glycosylation of a variety of proteins involved in hormone metabolism, such as transporters, receptors, and hormone processing could be the cause of hypogonadism and hypothyroidism in CDG syndromes.

This first section has aimed at providing an overview of the main endocrinopathies associated with a given IEM, summarized in Table 1. The next sections will aim to clarify which IEMs can be associated with a given endocrinological disturbance, such as diabetes (Table 2), dysthyroidism (Table 3) and hypogonadism (Table 4).

**Table 2:** Main causes of diabetes mellitus related to inborn errors of metabolism (IEM)

DISEASES	GENERAL CONTEXT	ENDOCRINE DYSFUNCTION: DIABETES	IEM DIAGNOSIS	TREATMENT
***Diabetes likely to signal an IEM: **rather insulinopaenic*				

**Hereditary haemochromatosis**	Melanodermy Hepatomegaly Rheumatologic involvement Early heart involvement in juvenile forms Complication: cirrhosis and hepatocarcinoma	**All types of Diabetes **10% - First insulin resistance - Then insulinopenia *Possible inaugural ketoacidosis* Hypogonadotropic hypogonadism	Transferrin saturation > 45%, serum ferritin > 200 (F), 300 (M) μg/l *HFE *gene mutation: HFE-1: 97% in Caucasians HFE-2: hemojuveline and hepcidin HFE-3: transferrin receptor HFE-4: ferroportin (autosomal dominant) Others	Insulin-sensitiser Insulin therapy Phlebotomy

**Hereditary acoeruloplasminemia**	Adult-onset neurological and psychiatric disease (chorea, cerebellar ataxia, retinal degeneration) Differential diagnosis of high blood ferritin *- Normal transferrin saturation* Dysmetabolic hepatosiderosis Inherited atransferrinaemia, Gaucher's disease -*Increased transferrin saturation* Multiple blood transfusions (thalassaemia)	**Diabetes mellitus **due to iron overload,	Anaemia High serum ferritin Absence of serum coeruloplasmin Coeruloplasmin gene mutations	Insulin therapy Iron chelation

**Mitochondrial diseases** **(respiratory chain defects)** MIDD MELAS Kearns-Sayre syndrome DIDMOAD	Deafness, pigmentary retinitis, Neuromuscular symptoms Kidney insufficiency Maternal inheritance	**Non-autoimmune diabetes in a lean patient > 30 years**, *possible inaugural ketoacidosis* Thyroid dysfunction More rarely hypoparathyroidism, adrenal insufficiency, hypogonadism, hypopituitarism Possible diabetes insipidus	Blood lactates/pyruvate ratio Blood ßOHbutyrate/acetoacetate ratio CSF lactates- urinary organic acids Plasma amino acids: high alanine and proline Muscle biopsy Mitochondrial DNA study Mitochondrial DNA or *WFS1 *gene study	Insulin therapy Coenzyme Q10

**Channelopathies **(*ABCC8 *gene)	Sometimes childhood fasting hypoglycaemia	**Early adult-onset non-autoimmune diabetes**	Heterozygous mutation in *ABCC8 *gene (autosomal dominant)	Sulfonylurea/insulin

***Diabetes complicating a previously diagnosed IEM***				

**Glycogen storage disease** **I and III**	Hepatomegaly (I and III) Mild muscular symptoms (Type III) Childhood fasting hypoglycaemia	**Progression to adult fasting hypoglycaemia with postprandial hyperglycaemia**	Fasting hypoglycaemia Lactic acidosis (preprandial I, postprandial III) Glc-6-P (I) and debranching enzyme (III) genes mutation	Alpha-glucosidase inhibitor and/or Insulin-sensitiser

**Alström syndrome**	Short stature Renal failure Dilated myocardiopathy Blindness, deafness	**Early insulin-resistant diabetes mellitus (82%)** Childhood obesity Hypogonadism Hypothyroidism	Major Hypertriglyceridaemia HypoHDLemia *ALMS I *gene mutation	Insulin-sensitiser Insulin therapy

**Cystinosis**	early-onset Fanconi syndrome with polyuria and hypophosphatemic rickets then complicated by blindness myopathy, central nervous system impairment- renal insufficiency.	**Diabetes mellitus 25%** Hypothyroidism 75% Hypogonadism 74% (males) Delayed puberty Growth retardation	Renal tubular Fanconi syndrome (hypokalemia, acidosis, dehydration, kidney loss of phosphate, glucose and amino acids)High leukocyte cystine Cystinosin (*CTNS*) gene mutatin	Electrolyte/vitamin supplementation Indomethacin Cysteamine

**Thiamine-responsive megaloblastic anaemia syndrome**	Megaloblastic anaemia Progressive perception deafness in infancy	**Thiamine-sensitive diabetes mellitus**, frequently insulin-dependent; possible ketoacidosis	*SLC19A2 *gene inactivation (thiamine transporter THTR1 gene)	B1Vitamin Insulin therapy

**Organic aciduria**	Cognitive disorders	**Transient hyperglycaemic ketoacidosis**	Urinary organic acids	Insulin therapy

The other genetic types of diabetes have been ruled out: Down's, Klinefelter, Turner, Friedreich, Huntington, Laurence-Moon-Biedl, Prader-Willi, porphyria, Toni-Debre-Fanconi, cystic fibrosis, Maturity Onset Diabetes of the Young (MODY) and neonatal diabetes. *Definitions*: F: female; M: male; MIDD: Maternal Inherited Diabetes Deafness; MELAS: Myopathy, Encephalopathy, Lactic Acidosis, Stroke; DIDMOAD: Diabetes Insipidus, Diabetes Mellitus, Optic Atrophy, and Deafness or Wolfram syndrome; Kearns-Sayre syndrome: Starting before 20 years with progressive ophthalmoplegia, retinitis pigmentosa, cardiac conduction disorders and multisystemic injury. Thiamine-responsive megaloblastic anaemia syndrome or Rogers syndrome. CSF: cerebrospinal fluid. G-6-P: Glucose-6-phosphatase.

**Table 3:** Main inborn errors of metabolism associated with thyroid dysfunction

DISEASES	CLINICAL MANIFESTATIONS	ENDOCRINE DYSFUNCTION: DYSTHYRODISM	DIAGNOSIS	TREATMENT
***Energy defect***				

**Mitochondrial disease**	Involving seemingly unrelated organs Maternal inheritance Deafness, retinitis pigmentosa Short stature, Neuromuscular symptoms Kidney insufficiency	**Goiter, hypo-/hyperthyroidism, thyrotropic insufficiency** Non-autoimmune diabetes in a lean patient > 30 years More rarely hypoparathyroidism, adrenal insufficiency, hypogonadism, hypopituitarism	Blood lactate/pyruvate ratio (high) Blood ßOH butyrate/acetoacetate ratio: high CSF lactate (high) Urinary organic acids Plasma amino acids: high alanine & proline Muscle biopsy Mitochondrial DNA study	Discuss coenzyme Q10

**Glycogenosis**	Liver involvement Infections in type Ib Growth failure Renal complications in adulthood	Increased prevalence of **autoimmune hypothyroidism in type I b** *Children: fasting ketotic hypoglycaemia* *Adults:* *I, III types*: polycystic ovary syndrome, diabetes, osteoporosis *VI, IX types*: spontaneous recovering delayed puberty	Fasting hypoglycaemia Hyper-lipaemia, -uricaemia Hyperlactataemia: - fasting predominant: type I - postprandial predominant: type III, VI, IX Leukocytes DNA gene mutation	Frequent food intake Uncooked cornstarch Low carbohydrate glycaemic index Night enteral feeding G-CSF in Ib type, Allopurinol Avoid oestroprogestative pills

***Degradation and synthesis of complex molecules***				

**Fabry disease**	Acroparesthesia Angiokeratoma Stroke at young age Renal, heart and eye involvement in boys	**Subclinical hypothyroidism** Isolated case report of subclinical adrenal insufficiency and hypoparathyroidism Infertility, osteoporosis	Alpha-galactosidase A in males Leukocytes *GLA *gene mutation in females (X-linked)	Substitutive recombinant enzyme therapy

**Cystinosis**	3 forms: infantile, juvenile, ophthalmic (adulthood) Liver and muscle involvement Evolution to end stage renal disease	**Hypothyroidism > 50%** Hypergonadotropic hypogonadism (in males) Insulin-dependant diabetes Growth failure	Gluco-phospho-aminic diabetes Hypokalaemia, acidosis, Leukocyte cystin measurement Leukocytes *CTNS *gene mutation	Electrolyte supplementation Vitamins Indomethacin Cysteamine

**Type 1 hyperoxaluria**	Recurrent oxalic lithiasis leading to end stage renal disease Bone, eye, heart involvement	**Hypothyroidism** - often severe - sometimes signalling disease	Hyperoxaluria Hyperglycoluria Alanine-glyoxylate-aminotransferase measurement on liver biopsy Leukocytes *AGXT *gene mutation	Abundant hydration Urine alkalinisation Pyridoxine phosphate Kidney and liver transplantation

**Neutral lipid storage disease**	Ichtiosis or Chanarin-Dorfman syndrome or myopathy/cardiomyopathy Hepatomegaly Central nervous system involvement	**Nodular dystrophy of the thyroid** (clear cells follicular adenoma)	Normal lipid levels CPK sometimes moderately increased Accumulation of triglycerides - in leukocytes (Jordan's abnormality) - in muscle, skin fibroblasts, liver (steatosis) Leukocytes or fibroblasts *ABHD5 *or *PNPLA2 *gene mutation	No effective treatment

**MCT8 deficiency** **Or Allan-Erndon-Dudley syndrome**	Severe cognitive deficiency, Hypotonia and dystonic movement Progressive spastic quadriplegia in boys	**High serum T_3 _- **low serum rT_3_ Low serum T_4 _(sometimes normal) Normal serum TSH (or slightly elevated) **Nodular dystrophy of the thyroid**	Leukocytes *MCT8 *gene mutation (X-linked)	Propylthiouracil and L- thyroxin

**Congenital disorders of glycosylation**	Affect nearly all organs and systems Often, significant neurological component.	**Most of the time difficulties in TSH measurement** *Always check with a reference method* *Rare congenital hypothyroidism* Hypogonadism	*N-glycosylation diseases*: serum transferrin isoelectrofocusing *O-glycosylation disorders*: apo CIII isoelectro-focusing, leukocytes DNA molecular study	Inhibitors of phosphomannose isomerase under evaluation in CDG-Ia Mannose in CDG-Ib

**Selenoprotein deficiency disorder**	Myopathy, Dermal photosensitivity	Deficiency of deiodinases: low serum T3 and and high serum T4 Oligospermia	Leukocytes *SECISBP2 *gene mutation	

***Intoxication disorders***				

**Haemochromatosis**	Liver, rheumatologic and heart involvement	**Hypo- and hyperthyroidism: ≤ 1%** Diabetes (10%)Peripheral hypogonadism (5-10%) Adrenal insufficiency, hypopituitarism, and hypoparathyroidism: similar to the general population (except for secondary iron overload)	Transferrin saturation Serum ferritin Leukocytes *HFE *gene mutation	Phlebotomy Iron chelation

*Abbreviations *3,3',5-triiodothyronine: T_3_; 3,3',5'-triiodothyronine: reverse T_3 _or rT_3_; tetraiodothyronine: thyroxine or T_4_; ATGL or Adipose triglyceride lipase, encoded by the gene *PNPLA2 or *patatin-like phospholipase domain containing 2; or alpha/beta-hydrolase domain-containing protein 5, encoded by *ABHD5 *gene (also called comparative gene identification-58); *AGTX *or Alanine-glyoxylate-aminotransferase gene; CDG or congenital disorders of glycosylation; *CPK: *creatin phospho kinase; CSF: cerebrospinal fluid; MCT8 (Monocarboxylate Transporter 8) deficiency or Allan-Erndon-Dudley syndrome or SLC16A2-Specific Thyroid Hormone Cell Transporter Deficiency; *SECISBP2 *or Sec insertion sequence-binding protein 2 (also known as SBP2)

**Table 4:** Main IEM associated with hypogonadism

DISEASES	CLINICAL MANIFESTATIONS	ENDOCRINE DYSFUNCTION: HYPOGONADISM	DIAGNOSIS	TREATMENT
***Intoxication diseases***				

**Haemochromatosis**	Melanodermy Hepatomegaly Rheumatologic signs Early heart involvement in juvenile forms Cirrhosis and hepatocarcinoma	**Hypogonadotrophic hypogonadism** - 5%-10% (HFE1)- early-onset in HFE-2 with hear involvementDiabetes 10% Hypo- and hyperthyroidism: ≤ 1% Adrenal insufficiency, hypopituitarism, and hypoparathyroidism: similar to the general population (except for secondary iron overload)	Transferrin saturation Serum ferritin Leukocytes *HFE *gene mutation	Phlebotomy Iron chelation Androgen with caution due to a possible increased risk of hepatocarcinoma

**Galactosaemia**	Mental retardation Cataracts Osteoporosis	**Hypergonadotropic hypogonadism**: 75-96%	Blood and urine measurement of galactose and galactitol Leukocytes *GALT *gene mutation	Galactose-free diet Oestrogen from 12-13 years; then + progestins Discuss recombinant FSH/oocyte cryopreservation Osteoporosis: Calcium ± vitamin D ± diphophonates

***Complex molecules disorders***				

**X-linked adrenoleukodystrophy**	Progressive central and peripheral nervous system demyelination in boys	**Clinical hypogonadism: 2/3** Erectile dysfunction: 58% Small testes: 12% Primary adrenal failure involving both gluco and mineralosteroid: 70% in adults	**Low testosterone12%** **Inadequate response to HCG: 88%** **High LH:16% and FSH levels: 32%** **High testosterone/DHT ratio** **High VLCFA** Leukocytes *ABCD 1 *gene mutation	Lorenzo oil Blood marrow transplantation Gene therapy

**Perrault syndrome or D-bifunctional protein deficiency**	Hearing loss Ataxia	Ovarian dysgenesis	Leukocyte *HSD17β4 *gene mutation, Sometimes mitochondrial DNA mutation	

**Congenital disorders of glycosylation** Most are protein hypoglycosylation diseases	Affect nearly all organs and systems Often a significant neurological component.	**Hypogonadism with small testes or amenorrhea** **Hypo-, hypergonadotropic or mixed hypogonadism** Sometimes hyperprolactinaemia Abnormal TSH Insulin resistance related to peculiar fat repartition	*N-glycosylation diseases:* serum transferrin isoelectrofocusing *O-glycosylation disorders:* apo CIII isoelectrofocusing Leukocytes gene mutation	Inhibitors of phosphomannose isomerase under evaluation in CDG-Ia Mannose in CDG-Ib

**Cystinosis**	3 forms: infantile, juvenile, ophthalmic (adulthood) Liver and muscle involvement Progression to end-stage renal disease	**Hypergonadotropic hypogonadism 74% in males **(gonadotropin levels above the normal range and testosterone levels in the low normal range); Delayed puberty Hypothyroidism > 50% Insulin-dependant diabetes Growth failure	Gluco-phospho-aminic diabetes Hypokalaemia, acidosis, Leukocyte cystin measurement *CTNS *gene mutation	Electrolyte supplementation Vitamins Indomethacin Cysteamine

**Fabry disease**	Acroparaesthesias in boys Angiokeratoma Stroke at young age Renal, heart and eye involvement	Hypothyroidism Isolated case report of subclinical adrenal insufficiency and hypoparathyroidism **Infertility**, osteoporosis	Alpha-galactosidase A in males *GLA *gene mutation in females (X-linked)	Substitutive recombinant enzyme therapy

**Alström syndrome**	Short height Renal failure Dilated myocardiopathy Blindness, Deafness	Early insulin resistant diabetes - Childhood obesity Primary hypogonadism (in males) PCOS and hirsutism in female Hypothyroidism	Major Hypertriglyceridaemia HypoHDLemia Leukocytes *ALMS I *gene mutation	

**Selenoprotein deficiency disorder**	Myopathy, Dermal photosensitivity	Oligospermia	low serum T3 and and high serum T4 Reduced selenoprotein concentrations Leukocytes *SECISBP2 *gene mutation	Selenium supplementation not efficient on hormone thyroid profile

***Energy defect***				

**Mitochondrial cytopathies**	*See *Table 2 *or 3* Maternal inheritance	Hypogonadotropic hypogonadism: 20% t- 30% delayed growth and puberty	*See Table 2 or 3*	*See Table 2 or 3*

**Glycogenosis**	Liver involvement Infections in type Ib Renal complications in adulthood	*Children: fasting ketosis hypoglycaemia*; growth failure *Adults: I, III types*: polycystic ovary syndrome, diabetes, osteoporosis *VI, IX types*: spontaneous recovering delayed puberty autoimmune hypothyroidism in type I b	hyperlipaemia, hyperlactataemia, hyperuricaemia Leukocytes gene mutation	Frequent food intake, uncooked cornstarch Low carbohydrate glycaemic index Night enteral feeding G-CSF in Ib type, allopurinol Avoid oestroprogestative pills

All autosomal recessive unless otherwise indicated

*Abbreviations: *DHT: Dihydrotestosterone; G-CSF: Granulocyte colony-stimulating factor; *GALT*: galactose-1-phosphate uridyltransferase; HSD17B4: 17beta-hydroxysteroid dehydrogenase type 4 (also known as D-bifunctional protein (DBP). PCOS: Polycystic ovary syndrome; VLCFA: Very long chain fatty acids.

## Diabetes

Several IEM can be associated with diabetes mellitus (Table 2). The main mechanisms of diabetes in IEM involve either defects of insulin secretion or insulin resistance, which might be promoted by liver or muscle involvement and estrogen deficiency [1]. The insulinopenic forms of diabetes are not associated with autoimmune features, and ketoacidosis might be the presenting sign, especially in haemochromatosis and mitochondrial diseases. In addition, the phenotype of some disorders might vary from hypo- to hyperglycemia according to age or genotype [defects in biotinidase (1-9/100000, ORPHA79241), organic acidurias, *ABCC8 *mutations].

### Diabetes likely to signal an IEM: Trend towards insulinopenia (Table 2)

***Hereditary haemochromatosis ***is the most frequent IEM in adults [2]. The phenotype varies according to the penetrance of the mutations of the *HFE 1 *gene, which affects 0.3% of the general population. The prevalence of diabetes in haemochromatosis, usually 20% to 50%, has decreased to 5% to 10% due to earlier genetic screening. In contrast, the prevalence of haemochromatosis in the diabetic population is 1.3%. Increased duodenal iron absorption due to an impairment of hepcidin synthesis accounts for the development of cellular iron excess in most cases of haemochromatosis. Genes other than *HFE1 *may account for hereditary forms of haemochromatosis. The form caused by mutations of the hemojuvelin gene has a juvenile onset and is often revealed by cardiopathy and hypogonadism. Non-transferrin-bound iron plays an important role in cellular iron damage through an increase in oxidative stress. First, liver iron overload promotes the occurrence of insulin-resistant diabetes [3]. The pancreas ß cells may then be progressively destroyed, leading to C-peptide negative diabetes and requiring insulin therapy besides iron depletion.

***Aceruloplasminemia ***(< 1-9/1000000, ORPHA48818) is characterized by the accumulation of iron in the liver, islets of Langerhans and the brain, related to decreased cellular iron egress, in contrast with most other types of iron overload [4]. The treatment with phlebotomies and iron chelation is efficient in preventing both neurological involvement and diabetes [5]. Annual glucose tolerance testing, starting at age 15 years, has been recommended.

***Mitochondrial diabetes ***makes up 0.06 to 2.8% of cases of type 2 diabetes and should be suspected in young, lean patients with non-autoimmune diabetes and neurosensory or muscular involvement [6-9]. It is maternally inherited and may be associated with multiple endocrine dysfunctions. Diabetes can progressively evolve towards insulin requirement, but inaugural ketoacidosis has also been reported. The need for insulin treatment is common, but the diabetes is usually easy to control. Proteinuria and renal insufficiency occur more frequently than usual in diabetes [10]. This form of diabetes is explained by insulinopenia, which is either related to the destruction of ß cells or to a defect of insulin secretion as a consequence of the energy defect. In the first case, the production of ATP through the glycolysis pathway, too low to ensure normal cell function, leads to cell death, as confirmed histologically by a reduction in the number of ß cells [11,12]. It also prevents closure of the potassium channels, inducing a defect in insulin secretion [13-15]. Insulin sensitivity in skeletal muscle is also decreased [15].

The most frequent genetic abnormality is the single mutation *A3243G *(mitochondrial DNA), particularly in MIDD (< 1-9/1000000, ORPHA225) and MELAS (< 1-5/10000, ORPHA550) syndromes. Heteroplasmy plays a central role in the phenotypic expression of the same mutation, and a higher level has been found in mitochondrial diabetes in the endocrine pancreatic ß cells than in the exocrine cells and blood leukocytes [16]. Correction of the consequences of mitochondrial 3243A > G mutation by tRNA import into mitochondria has been attempted, raising new hopes in the treatment of the disease [17].

Complex rearrangements are more often encountered in Kearns-Sayre syndrome (< 1-9/100000, ORPHA480), isolated diabetes and Wolfram syndrome (< 1-9/1000000, ORPHA3463) [18]. Wolfram syndrome, also called DIDMOAD (Diabetes Insipidus, Diabetes Mellitus, Optic Atrophy, Deafness) may also be linked to an autosomal recessive mutation of the *WFS1 *gene encoding Wolframin (nuclear DNA) [19]. In this case, diabetes appears earlier in males than in females, with low plasma insulin concentration and increased proinsulin/insulin ratio, which in the long-term may lead to insulin deficiency [20].

***Monogenic form of diabetes by ABCC8 gene mutations:*** Activating heterozygous mutations in *KCNJ11 *and *ABCC8 *genes, which form the ATP-sensitive K+ channel, usually causes transient or permanent neonatal diabetes. Nevertheless, the occurrence of diabetes in young adults has recently been reported and is related to a heterozygous activating mutation of the *ABCC8 *gene encoding the sulphonylurea receptor 1 (SUR1) [21]. The clinical phenotype of these patients is heterogeneous, either sensitive to sulfonylurea or requiring insulin. It appears to be modified by variable sensitivity to insulin according to hyperinsulinemic euglycemic clamp studies [22]. The phenotype associated with dominant inactivating (loss-of-function) *ABCC8/KCNJ11 *mutations, known to range from asymptomatic macrosomia to persistent hyperinsulinemic hypoglycemia in childhood, may also be an important cause of inherited early-onset diabetes mellitus in adults [23].

***In conclusion***, diabetes that is likely to reveal an IEM in adults is mainly related to hereditary haemochromatosis in association with hypogonadism, or to mitochondrial diabetes in association with multiple endocrinopathies. *ABCC8/KCNJ11 *mutations have been more recently identified in early-onset non-type 1 diabetes. The prognosis of hereditary haemochromatosis has improved due to earlier genetic screening.

### Diabetes complicating a previously diagnosed IEM (Table 2)

***Glycogen storage disease ***(GSD or glycogenosis) is the result of defects in the processing of glycogen synthesis or breakdown within muscles, liver, and other cell types. GSD is classified according to the type of genetically defective enzymes and induces hepatomegaly, hypoglycemia and/or muscular symptoms. GSD, especially types I (ORPHA364) and III (ORPHA366), can be complicated by diabetes in the later stage [24]. Type I GSD is initially associated with severe fasting hypoglycaemia. Postprandial hyperglycemia (sometimes in contrast with fasting hypoglycaemia) can occur in adulthood [25]. This fact could be explained either by recurrent pancreatitis episodes linked to hypertriglyceridemia, thus leading to insulin secretion impairment, or to an adaptive mechanism of the glucose receptor, GLUT2, for reducing the secretion of insulin. Diabetes is also promoted by insulin resistance related to liver and/or muscle dysfunction [26,27]. Gene therapy is under evaluation.

***Alström syndrome:*** Alström syndrome (< 1-9/1000000, ORPHA64) is characterized by a wide-ranging spectrum of phenotypes. ^.^In a series of 182 patients, hyperinsulinemia developed in early childhood (92%) and progressed to type 2 diabetes mellitus in 82% of those older than 16 years. Hypertriglyceridemia (54%) precipitated pancreatitis in 8 patients [28]. An oral glucose tolerance test should be considered annually, since fasting blood glucose is often normal at diagnosis. If blood glucose control is poor on insulin therapy, escalating doses may not be effective [29]. Unlike insulin-resistance indices, β-cell function indices show a significant reduction with age [30]. Mutations of the *ALMS1 *gene cause dysfunction of the primary cilium, an organelle involved in cell sensing, as in another model of genetic obesity, the Bardet-Biedl syndrome. The higher frequency of diabetes in Alström syndrome is explained by the specific role of *ALMS1 *in β-cell function and/or peripheral insulin signaling pathways, in parallel with adipogenesis impairment [31].

***Cystinosis ***is a lysosomal disease, which induces an intracellular accumulation of cystine due to transportation impairment. It presents as a multisystemic injury dominated by renal manifestations. Twenty-four percent of a cohort of 100 patients had diabetes, with a quarter of those needing insulin therapy, in addition to other endocrinopathies such as hypothyroidism and hypogonadism [32-34]. The pathophysiology of diabetes mellitus is mainly due to decreased insulin secretion associated with pancreatic fibrosis. Microarray studies have shown that some of the differentially regulated genes in cystinosis were involved in mitochondrial dysfunction, endoplasmic reticulum and oxidative stress, as well as immune function [35].

***Thiamine-sensitive megaloblastic anemia (Rogers syndrome) ***[36] is characterized by diabetes and deafness due to a lack of thiamine in pancreatic islet and cochlear cells. It is caused by a defect in the active transport of thiamine (thiamine transporter 1 or THTR1), which leads to ß cell apoptosis and insulin secretion impairment. In contrast with neurosensory disorders, anemia and diabetes improve with pharmacological doses of oral thiamine and may lead to insulin discontinuation. Poor blood glucose control however reoccurs most of the time during adolescence, thus requiring resumption of insulin therapy [37,38].

***Organic Aciduria:*** Propionic (< 1-9/100000, ORPHA35), methylmalonic (< 1/1000000, ORPHA26) and isovaleric acidemias (1-9/100000, ORPHA33) are sometimes discovered during infancy through the presence of hyperglycemic ketoacidosis, which may suggest diabetes, particularly during pancreatitis, one of the complications of the disease [39,40]. Diabetes has never been reported to be a presenting manifestation of organic aciduria in adulthood, but should be kept in mind since mitochondrial dysfunction has been reported to be associated with it [41].

***Finally***, diabetes may also complicate a previously diagnosed IEM, particularly those inducing impairment of the degradation or synthesis of complex molecules. In this group, hypothyroidism or hypogonadism are frequently associated endocrine disorders.

**In conclusion**, diabetes, mainly linked to ß cell dysfunction, is encountered in the 3 types of IEM: intoxication disorders (mainly haemochromatosis, sometimes organic aciduria), disorders of energy metabolism (respiratory chain defects, GSD) or organelle disorders (Alström and Rogers syndromes, cystinosis). Of these IEM, iron overload has a special place in adulthood since hereditary haemochromatosis linked to a mutation of the *HFE 1 *gene is one of the most frequent metabolic diseases, even if its penetrance is variable and is modulated by environmental factors such as body weight and alcohol.

## Dysthyroidism

The IEMs associated with dysthyroidism in adulthood mainly involve energy metabolism defects and the degradation or synthesis of complex molecules (Table 3). Primary hypothyroidism is the most frequent feature, although pituitary participation cannot be excluded in most cases. Goiter has also been reported. Hyperthyroidism is more questionable. Besides the consequences of dysthyroidism, hypothyroidism can affect reproduction and glucose metabolism. When a pregnancy is possible, preconceptional FT4 and TSH should be assessed, taking into account the known influence of even subclinical hypothyroidism on early fetal brain development and long-term cognitive function [42]. Otherwise, overt hypothyroidism has been associated with increased rates of spontaneous abortion, premature delivery and/or low birth weight, fetal distress in labor, and perhaps gestational hypertension, emphasizing the importance of thyroid balance before and during pregnancy [43]. Hypothyroidism may also induce anomalies in ß cell development, showing the interrelations between the different hormonal axes [44].

### Energy metabolism defects

***Mitochondrial cytopathies***, especially Kearns-Sayre and MELAS syndromes, have been reported to be the cause of primary dysthyroidism in several publications, three cases of which were autoimmune hyperthyroidism [8,9,45-48]. Given the frequency of autoimmune dysthyroidism in the general population, a fortuitous association between mitochondrial cytopathies and thyroidopathies cannot be ruled out. Indeed, *A3243G *mitochondrial mutation does not actually occur significantly more often in patients with autoimmune diseases. We personally observed one-third of cases with hypothyroidism, 50% of which displayed a goiter or a pituitary component in a series of respiratory deficient patients (unpublished observation). Testing should always be done for both origins. Thyrotropic insufficiency is probably underestimated. Energy defects could impair thyroperoxidase, lowering the production of thyroid hormones, which themselves are involved in mitochondrial metabolism, therefore worsening the mitochondrial dysfunction.

***Glycogen storage disease:*** An increased prevalence of autoimmune hypothyroidism has been found in type Ib glycogenosis (glucose-6-phospate translocase defect) (ORPHA79259); however, patients with the type Ia form (glucose-6 phosphatase defect) (ORPHA79258) had a low frequency of thyropathies in a series of patients with glycogenosis (10 with type 1a and 7 with type Ib) compared to 34 matched controls [49]. FT4 blood levels were significantly lower in both type Ia and Ib than in the control group, whereas TSH, thyroglobulin levels and antithyroperoxidase antibodies were higher only in patients with type Ib. Central impairment may be associated with these anomalies, since the increase of TSH level always remains mild. Indeed, glucose-6-phosphatase defect inducing glycogen accumulation has been observed in the pituitary, adrenal, thyroid, parathyroid, and pancreatic glands. Otherwise, most GSD Ib patients also show neutropaenia and therefore are at risk of developing infections and autoimmune-related disorders.

### Degradation or synthesis of complex molecules

***Fabry disease ***is an X-linked lysosomal disease related to an alpha-galactosidase A deficiency, which induces an accumulation of globotriaosylceramide. Mild forms of the disorder may appear later in life and affect only the heart or kidneys. Subclinical, non-autoimmune hypothyroidism was observed in 4/11 cases in an untreated series, and in 3 cases associated with adrenal and gonadal disturbances in another series of 18 patients, 10 of whom were being treated with substitutive enzyme therapy [50,51]. Osteopenia was frequent. Subclinical primary hypothyroidism has been shown to improve after long-term enzyme replacement therapy [52].

***Cystinosis ***was complicated by hypothyroidism in 75% of patients [32-34].

***Type 1 hyperoxaluria (< 1-9/1000000, ORPHA93598) ***is induced by a liver peroxisomal deficiency secondary to alanine glyoxylate aminotransferase gene mutations [53]. Several cases of hypothyroidism revealing or complicating this renal disease have been reported, without any significance as to the age of the patient [54,55]. Hypothyroidism is usually severe, probably linked to the tissular accumulation of calcium oxalate, but also to immunoreactivity deficiency and sensitivity to proteasomal degradation induced by impaired dimerization in the mutated gene variants.

***Neutral lipid storage disease (< 1/1000000, ORPHA155): ***This rare non-lysosomal lipid storage disorder is caused by defects in two triglyceride-associated proteins: adipose triglyceride lipase, encoded by the gene "patatin-like phospholipase domain-containing 2" (***PNPLA2***); and "alpha/ß-hydrolase domain-containing protein 5", encoded by the *ABHD5 *gene, also called "comparative gene identification-58". Dysfunction of these two proteins affects the degradation of triglycerides, and then causes their accumulation in cells with two different phenotypes [56]. Thyroid nodular dystrophy related to intracellular accumulation of triglycerides has been reported in lipid storage disease with the "ichtyosis" phenotype [57] (see Table 3).

***MCT8 deficiency:*** This X-linked mental retardation syndrome in male subjects involves the transport of triiodothyronine into neurons with high T_3 _and inadequately low T4 and fT4 concentration [58]. This biological effect persists after complete thyroidectomy and substitution with levothyroxine, which involves peripheral deiodination. Progressive thyroid follicular dystrophy with a possible risk of papillary carcinoma has been recently reported [59]. Heterozygous *MCT8 *women, usually asymptomatic, should be monitored for the requirement of L-T4 therapy to prevent fetal and neonatal hypothyroidism [58,60]. In families at risk, a prenatal diagnosis for male fetuses can be done. Treatment with propylthiouracile and L-thyroxine has been proposed [61].

***Congenital disorder of glycosylation:*** Thyroid function tests are frequently abnormal in children with CDG; congenital hypothyroidism has been suspected. However, free thyroxine analyzed by equilibrium dialysis, the most accurate method, was reported as normal in seven individuals with CDG [62]. In contrast, CDG are associated with hypogonadism and are detailed in this section.

***Selenoprotein deficiency disorder (ORPHA209193):*** Selenium is a micronutrient involved in selenoprotein metabolism, such as the glutathione peroxidase, mediating the removal of cellular reactive oxygen species, and deiodinases, which are involved in thyroxine metabolism. Mutations in the *SECISBP2 *(Sec insertion sequence-binding protein 2, also known as SBP2) gene result in reduced synthesis of all selenoproteins, and have a multisystem expression with an abnormal thyroid hormone profile (low T3, high T4) [63,64]. Selenium supplementation fails to correct the selenoprotein synthesis defect in subjects with SBP2 gene mutations [65].

***Haemochromatosis:*** A case of a 56-year-old male patient with haemochromatosis complicated by cirrhosis, insulin-dependent diabetes and hypogonadism with regressive hypothyroidism after venopuncture has recently been reported [66]. Nevertheless, in two studies of 100 to 200 homozygote *C282Y *patients, the prevalence of hypo- and hyperthyroidism that was below 1% did not differ from that of the control group [67]. Dysthyroidism was however more frequent in acquired haemochromatosis.

**In conclusion**, dysthyroidism may complicate energy metabolism diseases (respiratory chain defect, GSD) or organelle disorders affecting mainly the kidney (cystinosis, type I hyperoxaluria) or selectively the thyroid gland (MCT8 and selenoprotein deficiencies).

## Hypogonadism

Medical advances have enabled the survival of patients suffering from IEM. Nevertheless, hypogonadism remains one of the most frequent complications in adolescence. Optimization of pubertal progress should reduce the impact of growth retardation, later adult bone loss and related psychosocial issues. Screening for primary or hypogonadotrophic hypogonadism, as well as osteoporosis, should be done in adulthood.

### Intoxication disorders

***Haemochromatosis:*** Hypogonadotropic hypogonadism was found in 5.2% of a series of 38 female patients and 6.4% of a series of 141 male subjects, 89% of whom also had cirrhosis and 33% diabetes [68]. This prevalence of hypogonadism had been previously evaluated at 40%, with this fact being linked to an earlier diagnosis and treatment. In another series of 87 patients with hereditary hemochromatosis, osteoporosis was detected in 25% and osteopenia in 41%. Bone mineral density was independently associated with body mass index, hypogonadism/menopause, and the amount of iron removed to reach depletion [69]. Furthermore, haemochromatosis should always be suspected in male subjects with isolated hypogonadotropic hypogonadism. In young men, the diagnosis of juvenile haemochromatosis, despite its rarity, should be considered, especially in case of cardiac involvement. At least three Japanese men bearing new mutations of the *HJV *(hemojuveline) gene, which is usually found in so-called "juvenile" haemochromatosis, were diagnosed in their fifties due to hypogonadism, showing the wide variability of the phenotype [70]. Pituitary iron overload, as well as the *HFE *and transferrin genotype, may explain the hypothalamic-pituitary-gonadal involvement [71]. If diagnosed early, hypogonadism can regress after phlebotomy. The treatment with androgens might be difficult considering the increased risk of hepatocarcinoma when associated with cirrhosis and the potential carcinogenic role of androgen therapy [72].

***Classical galactosemia ***is a not so rare disorder of galactose metabolism. Despite a strict diet and newborn screening, long-term complications usually include mental retardation, cataracts and ovarian insufficiency in most girls [73]. Indeed, in a series of 34 female patients aged 17 to 51 years, only 4 menstruated regularly without any treatment, and a single pregnancy occurred spontaneously [74]. In another series of 33 patients (16 women), all female subjects had ovarian insufficiency. One woman and two men had children. The genotype does not seem to predict the chances of becoming pregnant [73,74]. During pregnancy, elevations in galactose metabolites do occur but without evidence of clinical impact on the mother or child, although possible long-term effects have not been thoroughly investigated [75].

Many mechanisms are involved including: 1) an accumulation of galactose-1-phosphate, which induces a rapid loss of follicles through cellular apoptosis, and of galactitol, which alters the ovarian tissue; and 2) a deficiency in UDP-galactose, which alters glycosylation, possibly impairing FSH activity [76-79]. Hypoglycosylation is diet-dependent and may worsen when galactose intake increases either because of poor compliance or unrestricted diet.

***Currently***, haemochromatosis and galactosemia are the two main intoxication diseases that are complicated by hypogonadism. In contrast with iron overload, in which hypogonadism can be prevented or improved through treatment, hypogonadism in female patients with galactosemia is a unique endocrine complication and occurs in most patients despite early prospective treatment.

### Organelle disorders involving the synthesis or degradation of complex molecules

***X-Adrenoleukodystrophy (X-ALD) and D-bifunctional protein (D-BF) deficiency (ORPHA300):*** X-ALD is a progressive peroxisomal disorder affecting adrenal glands, testes and myelin stability caused by mutations in the *ABCD1 *gene, which encodes for ALD protein. The anomalies of this peroxisomal transporter induce a ß-oxidation defect of VLCFA [80,81]. X-ALD is associated in more than two-thirds of cases with clinical hypogonadism and anomalies of the gonadotropic axis [82]. Severe impairment of spermatogenesis and rapid progression to azoospermia were reported despite normalization of plasma VLCFA concentrations in a post-pubertal patient [83]. Oxidative stress plays a role, at least in the neurodegeneration features [84]. Otherwise, although D-BF protein deficiency is in general a severe neonatal or infantile disease mimicking the peroxisome biogenesis or so-called Zellweger spectrum disorders [85,86], two siblings with Perrault syndrome were reported with mutations in the D-BP (*HSD17β4) *gene [87]. However, other patients with Perrault syndrome had no mutations in this gene, indicating that this syndrome is genetically heterogeneous and is sometimes related to mutations of mitochondrial DNA [88].

***Congenital disorders of glycosylation (CDG) ***are a rapidly growing group of diseases that impair protein glycosylation, one of the major functions occurring in the endoplasmic reticulum and Golgi compartments [89]. The clinical manifestations are heterogeneous [90,91]. Elevated tissue fibrosis, frequent in CDG patients, could promote hypogonadism, which is frequent, especially in males [92-97]. Varying hormonal profiles and degrees of virilization in CDG females suggest a spectrum of yet unidentified mechanisms affected by impaired N-glycosylation [98]. Potential treatment of the most common form, CDG-Ia, with phosphomannose isomerase inhibitors is under evaluation [99].

***Cystinosis:*** The onset of puberty was consistently delayed in a series of 17 patients with chronic renal insufficiency [100]. Hypergonadotropic hypogonadism has been found in 50% to 75% of males. It seems to be related to fibrosis and testicular atrophy and promotes growth retardation [33,34,100].

***Fabry disease:*** 89% of women may have menstrual disorders (or spontaneous abortions), although gonadotropic stimulation tests were normal in women; oligo/asthenozoospermia is frequently found in males [51,101].

***Alström disease:*** The most common endocrinological manifestation besides type 2 diabetes is hypogonadism. In a series of 182 patients (83 males) [28], hypergonadotropic and hypogonadotropic hypogonadism were common in males (77%). Puberty was often delayed, but masculinization and secondary sexual characteristics in adult males were normal. Basal testosterone levels were frequently diminished for age with increased baseline luteinizing hormone/follicle-stimulating hormone levels typical of primary gonadal failure. Gynecomastia was present in 37% of males, as was cryptorchidism in 0.02% of cases. The mean menarchal age was 12.6 years. Endocrine disturbances were observed in 54% of 78 females and included hirsutism, abnormal breast development, cystic ovaries, precocious puberty (pubertal onset at age < 8 years), endometriosis, irregular menses and amenorrhea.

***Finally***, peroxysomal, lysosomal and reticulum endoplasmal disorders are frequently associated with manifestations such as delayed onset of puberty, hypogonadism and infertility, as well as other endocrine disorders. Their responsiveness to treatment has not been fully investigated up till now.

### Energy metabolism disorders

***Mitochondrial cytopathies:*** Hyper- [88,102] and hypogonadotropic [9,103-105] hypogonadism have been described in case reports [106], but the frequency can reach 20% in patients with Kearns-Sayre syndrome [45]. Anomalies of estrogen metabolism in the muscle have also been reported in these patients who often have a myopathic component [107,108].

***In a series with glycogen storage disease***, both type I (n = 13) and type III (n = 14), all patients older than 4.8 years presented with polycystic ovary syndrome but without hormonal biological disturbances, except hyperinsulinaemia [109]. Delayed puberty-related growth impairment and short stature have also been described. The role of glucose metabolism alteration is probably involved in the ovarian pathophysiology.

***Finally***, hypogonadism is a less frequent complication of energy metabolism disorders than of organelle disorders.

**In conclusion with regard to hypogonadism**, most IEMs can be complicated by hypogonadism, but classical galactosemia and organelle disorders are the most frequently involved diseases. Except for iron overload, in which hypogonadism might be prevented or improved with iron depletion, the mechanisms are not yet fully understood and causal treatment remains difficult.

## Adrenal failure

As with thyroid hormones, steroids are liposoluble hormones, and adrenal insufficiency has mainly been reported in adrenoleukodystrophy (X-ALD) and energy metabolism defects.

***X-*adrenoleukodystrophy**, the most common cause of primary adrenal failure in IEM, is associated with neurological symptoms [80,81]. In adulthood, adrenal insufficiency, which generally appears after the age of 3-4 years, is present in 70% of patients. It can be the first and only manifestation of the disease for decades. Its association with neuropathy suggests the diagnosis. At age 20 years, it is usually associated with hypergonadotropic hypogonadism.

***Fabry disease:*** Patients with Fabry disease have been found to have lower mean cortisol plasma levels than in controls. Partial adrenal insufficiency was observed after the ß1-24 corticotrophin test in only one of the 18 studied patients [51]. These data need to be confirmed since acute adrenal failure has never been reported before.

***Niemann-Pick disease (< 1-9/100000, ORPHA645) ***is a lysosomal lipid storage disease caused by mutations in the *sphingomyelin phosphodiesterase 1 *(*SMPD1) *gene, which results in the deficient activity of lysosomal acid. Partial adrenal insufficiency has been reported in type B, a non-neurologic, visceral form with hepatosplenomegaly, pulmonary disease and survival into adolescence and/or adulthood [110].

***Mitochondrial cytopathies:*** Adrenal failure is rare in childhood but might reveal the presence of the disease [111], thus being a poor prognostic factor [112-114]. In adulthood, subclinical adrenocortical insufficiency is sometimes found in multisystemic forms [115]. Various types of aldosterone secretion disturbances, mainly secondary hyperaldosteronism possibly linked to tubulopathies, have also been reported [45,116].

## Hypoparathyroidism

Hypoparathyroidism may occur, though rarely, in all types of IEM, the most frequent being ***mitochondrial cytopathies ***[117-119].

Hypoparathyroidism might also complicate acquired ***iron overload in major thalassemia ***[120], and occasionally ***adrenoleukodystrophy, and Fabry or Wilson's disease (< 1-9/100000, ORPHA905)***, a congenital disorder of copper metabolism affecting the hepatic and nervous systems [121,122].

In ***fatty acid oxidation (FAO) disorders***, and particularly in LCHAD (long-chain 3-hydroxyacyl-coenzyme A dehydrogenase) deficiency (< 1-9/100000, ORPHA5) and trifunctional enzyme defects, which are characterized by neuropathy and retinopathy, transient hypoparathyroidism occurs frequently in cases of acute decompensation and is usually asymptomatic [123,124]. Later-onset exercise-induced myopathic symptoms remain characteristic clinical features of FAO disorders, in addition to heart and liver involvement.

## Hypopituitarism and growth

Pituitary assessment has not been systematically done in IEM. Relative problems could go partially unrecognized due to the difficulty of the evaluation in subclinical forms and the possible mixed primary and pituitary involvement. Otherwise short stature might also be multifactorial due to liver or renal dysfunction, poor nutritional status or psychosocial issues.

A few cases of ***hypopituitarism and diabetes insipidus ***[19] have been reported in mitochondrial cytopathies [9,45,103,104] and iron overload [125-127].

***Growth retardation ***was found in 30% to 60% of mitochondrial cytopathies, cystinosis [34] and galactosemia [128]. The resulting short stature is also found in Niemann-Pick disease [110] and Alström syndrome [28].

However, Alström syndrome is associated with advanced bone age, and normal early growth may be due to hyperinsulinism [28,129,130], although in a series of 15 patients, no significant relation was observed between IGF (Insulin Growth Factor) levels and body mass index or blood glucose, insulin and testosterone levels [131]. Type 1 hyperoxaluria is also associated with advanced skeletal age in young patients, increased FGF23 (Fibroblast Growth Factor 23) levels and decreased bone mineral density, thus promoting fractures [132]. Further investigations, including GH (Growth Hormone) dynamics, are needed to determine whether disturbances in the GH/IGF axis contribute to this relatively short stature, the origin of which is probably multifactorial through decreased renal function, feeding difficulties, hypothyroidism, hypogonadism, etc.

## Conclusion

With the exception of iron overload, IEM are very rare disorders. They may be associated with endocrine disorders, most of which are linked to carbohydrate metabolism disturbances, thyroid dysfunction and hypogonadism. The endocrinologist may screen patients with a diagnosis that was established in childhood for complications. In rarer cases, the diagnosis of IEM should be suspected when there are multiple endocrinopathies or when there is associated multisystemic injury. A prospective investigation of endocrine function should probably be scheduled in IEM, since numerous asymptomatic endocrine disorders with delayed manifestations have been found. Indeed, the long-term consequences of IEM on fertility and bone are still poorly understood. Mechanisms such as energy required for hormonal synthesis, toxic accumulation of a metabolite, hormone transportation defect or hormone/receptor glycosylation should be better investigated in order to improve our understanding of the pathophysiology and to help innovate in the screening and treatment of endocrine disorders. A few biological exams relying on baseline blood and urine sampling can steer the diagnosis. Consultation with a specialist is usually required in reference centers, with whom it is often necessary to collaborate.

## List of abbreviations

ABHD5: alpha/ß-hydrolase domain-containing protein 5; AGTX: Alanine-glyoxylate-aminotransferase gene; ATGL: Adipose triglyceride lipase; CDG: congenital disorders of glycosylation; CPK: creatin phospho kinase; CSF: cerebrospinal fluid; DBP: D-bifunctional protein; DHT: Dihydrotestosterone; DIDMOAD: Diabetes Insipidus, Diabetes Mellitus, Optic Atrophy, and Deafness; FGF23: Fibroblast Growth Factor 23; GALT: galactose-1-phosphate uridyltransferase; G-CSF: Granulocyte colony-stimulating factor; GH: Growth hormone; G-6-P: glucose-6-phosphatase

GSD: glycogen storage diseases; HSD17B4: 17ß-hydroxysteroid dehydrogenase type 4; IEM: inborn errors of metabolism; IGF: Insulin Growth Factor; LCHAD: long-chain 3-hydroxyacyl-CoA dehydrogenase deficiency; MCT8: Monocarboxylate Transporter 8; MELAS: Myopathy, Encephalopathy, Lactic Acidosis, Stroke; MIDD: Maternal Inherited Diabetes Deafness; MODY: Maturity Onset Diabetes of the Young; PCOS: polycystic ovary syndrome; PNPLA2: patatin-like phospholipase domain containing 2; reverse T3 or rT3: 3,3',5'-triiodothyronine; SECISBP2: Sec insertion sequence-binding protein 2; SMPD1: sphingomyelin phosphodiesterase 1; T3: 3,3',5-triiodothyronine; T4 or thyroxine: tetraiodothyronine; VLCFA: Very long chain fatty acids; X-ALD: X-linked adrenoleukodystrophy

## Competing interests

The authors declare that they have no competing interests.

## Authors' contributions

MCV, CD and JMS wrote and coordinated the writing of the manuscript; KM, DD and JLW participated in the design of the review and helped to draft the manuscript. All the authors read and approved the final manuscript.
